# Integrating Nutrition, Inflammation, and Immunity: The CALLY Index as a Novel Prognostic Biomarker in Acute Geriatric Care

**DOI:** 10.3390/nu17203192

**Published:** 2025-10-10

**Authors:** Francesca Mancinetti, Anna Giulia Guazzarini, Martina Gaspari, Michele Francesco Croce, Rocco Serra, Patrizia Mecocci, Virginia Boccardi

**Affiliations:** 1 Division of Gerontology and Geriatrics, Department of Medicine and Surgery, University of Perugia, 06123 Perugia, Italyannagiuliaguazzarini@gmail.com (A.G.G.); patrizia.mecocci@unipg.it (P.M.); 2 Division of Clinical Geriatrics, Department of Neurobiology, Care Sciences and Society, Karolinska Institutet, 17177 Stockholm, Sweden

**Keywords:** geriatric risk stratification, malnutrition, inflammation, prognostic biomarker, C-reactive protein–albumin–lymphocyte index

## Abstract

**Background/Objectives:** Malnutrition, systemic inflammation, and immune dysfunction are key determinants of adverse outcomes in older adults following acute illness. Composite biomarkers integrating these domains could enhance early risk stratification. This study investigates, for the first time in acute geriatric care, the prognostic value of the C-reactive protein–albumin–lymphocyte (CALLY) index—a composite marker of nutritional, inflammatory, and immune status—in predicting short-term survival. **Methods:** We retrospectively analyzed 264 patients admitted to the acute geriatrics ward of Santa Maria della Misericordia Hospital in Perugia. The CALLY index was calculated as: (Albumin × Lymphocytes)/(CRP × 10^4^). The optimal prognostic cut-off was determined using receiver operating characteristic (ROC) curve analysis. Three-month survival was assessed by Kaplan–Meier analysis. **Results:** The cohort included 167 women (63.3%) and 97 men (36.7%), with a mean age of 88.0 ± 6.4 years. At 3-month follow-up, 80 patients (30.3%) had died. The CALLY index showed an area under the ROC curve of 0.647 (95% CI: 0.576–0.718; *p* < 0.001), with a cut-off of 0.055 (sensitivity: 68.5%, specificity: 46.3%). Among deceased patients, 42.5% had a CALLY index <0.055. After multivariable adjustment, a lower CALLY index remained independently associated with increased mortality (B = −0.805; OR = 0.45; 95% CI: 0.215–0.930; *p* = 0.031). Kaplan–Meier analysis demonstrated significantly higher survival in patients with a CALLY index ≥ 0.055 (Log-rank test: 13.71; *p* < 0.001). **Conclusions:** The CALLY index shows a modest but statistically significant discriminative ability for predicting short-term mortality in acutely ill older adults. As a simple, low-cost marker derived from routine laboratory tests, it holds potential for integration into clinical workflows to guide nutritional, metabolic, and prognostic management strategies in geriatric acute care.

## 1. Introduction

The identification of reliable prognostic markers in acutely ill older adults is a major challenge in geriatric medicine. Multimorbidity, functional decline, and age-related changes often complicate outcome prediction after hospitalization [1]. Among prognostic factors, malnutrition and systemic inflammation are highly prevalent and strongly linked to adverse outcomes [2]. Composite indices combining nutritional and inflammatory biomarkers have therefore gained attention for risk stratification. The CALLY index combines three complementary components—C-reactive protein, serum albumin, and lymphocyte count—into a single biomarker that reflects the interplay between nutritional status, systemic inflammation, and immune competence. As such, the index provides an integrated view of biological resilience, capturing dimensions of vulnerability that are often under evaluated when these parameters are assessed individually [3]. Originally validated in oncology [4,5], the CALLY index has demonstrated prognostic value in various clinical scenarios, including sepsis [6], cardiovascular diseases [7], and acute ischemic stroke [8]. For instance, studies have shown that a lower CALLY index is associated with higher mortality in sepsis patients, and it serves as an independent predictor of poor functional outcomes in acute ischemic stroke patients undergoing endovascular thrombectomy [6,9,10]. While the prognostic utility of the CALLY index has been explored in these settings, its potential role in acute geriatric care remains unexplored. Older adults admitted for acute medical conditions represent a distinct population with pathophysiological profiles that differ substantially from younger or disease-specific cohorts. Identifying reliable biomarkers in this setting is therefore critical to improving early risk stratification and guiding individualized interventions. Recent studies performed in community-dwelling older adult populations suggest that the CALLY index may serve as a practical prognostic biomarker for monitoring survival [11]. Thus, to address this knowledge gap, the present study aimed to investigate, for the first time, the prognostic significance of the CALLY index in very old persons admitted to an acute geriatric care unit. By integrating routine laboratory data into a single composite marker, we sought to assess whether the CALLY index could serve as a practical, low-cost tool to support early risk stratification and clinical decision-making in acute geriatric practice.

## 2. Materials and Methods

### 2.1. Subjects and Study Design

**Figure 1:**
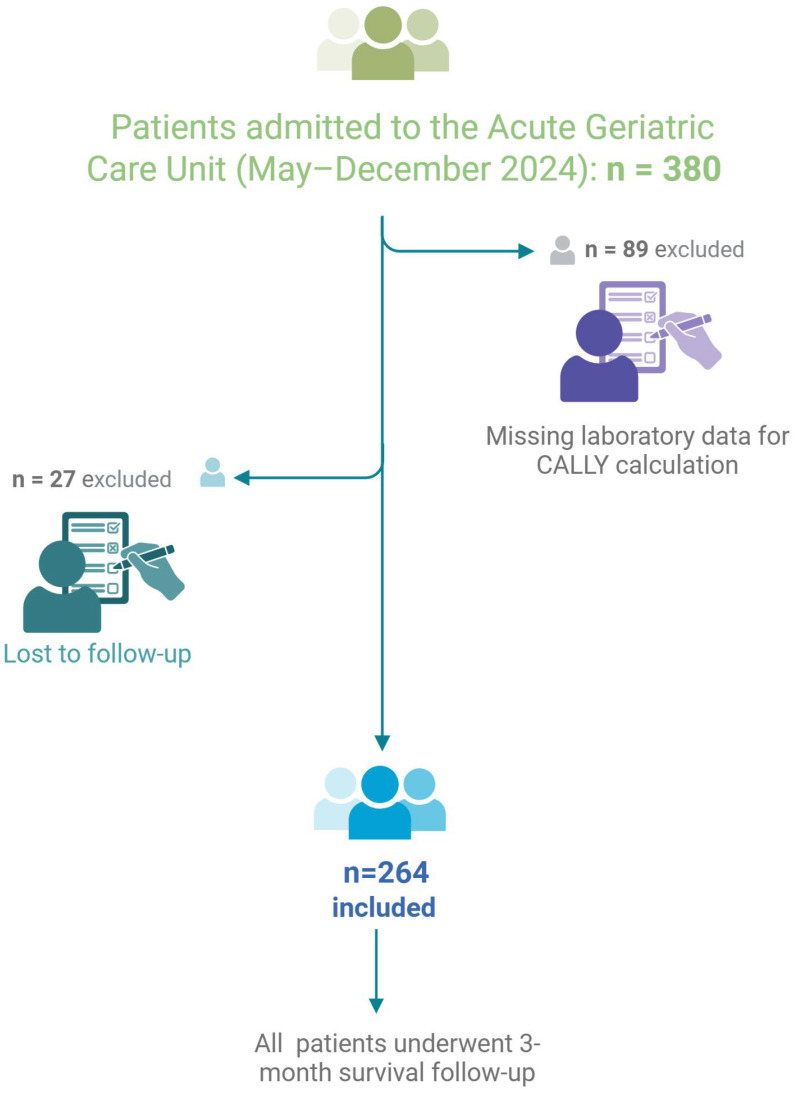
Flowchart of patient selection and study cohort. A total of 380 patients admitted to the hospital between May and December 2024 were screened for eligibility. Of these, 116 were excluded due to missing laboratory data required for CALLY index calculation (*n* = 89) or loss to follow-up (*n* = 27). The final cohort comprised 264 patients, all of whom were included in the analysis and underwent three-month survival follow-up.

This was a retrospective, observational, single-center study conducted at the acute Geriatric Care Unit of Santa Maria della Misericordia Hospital in Perugia, Italy, between May and December 2024. The study population included individuals aged 65 years and older admitted to the unit during this period. Eligibility criteria required that participants undergo routine laboratory assessments including complete blood count, C-reactive protein (CRP), and serum albumin—parameters essential for calculating the CALLY index. Additionally, participants needed to be reachable for a structured telephone follow-up three months after hospital discharge. Patients who were unable to provide informed consent were excluded from the study. Of the 380 individuals initially screened, 264 were included in the final analysis. Patients were excluded if any of the laboratory values necessary for CALLY index calculation (serum albumin, lymphocyte count, or CRP) were missing or if follow-up information could not be obtained (Figure 1). A three-month survival endpoint was chosen as it captures short-term outcomes that are clinically meaningful in acute geriatric care, allowing sufficient time for post-discharge complications to manifest while minimizing confounding from unrelated long-term events.

The research protocol adhered to the ethical standards set forth in the Declaration of Helsinki and followed the principles of Good Clinical Practice. Ethical approval was obtained from the Ethics Committee of the University of Perugia (Protocol No. CE-685/24).

### 2.2. Clinical and Multidimensional Assessment

Clinical information was obtained by clinical history and geriatric multidimensional evaluation. The main vital parameters such as blood pressure according to the Riva-Rocci method [12] and peripheral oxygen saturation by use of a pulse oximeter. The geriatric multidimensional evaluation was complemented by the administration of scales that explores different domains including functional status through the Activities of Daily Living (ADL)/Instrumental Activities of Daily Living (IADL) scales [13], nutritional status through the Mini Nutritional Assessment (MNA) scale [14], the presence of comorbidities through the Cumulative Illness Rating Scale (CIRS-G) [15], and polypharmacy therapy. The Sequential Organ Failure Assessment (SOFA) was used to identify the severity of the patient’s clinical condition at the time of hospital admission. A simplified score based on the SOFA score is the qSOFA score, which is said to be more accurate than SOFA in departments outside the intensive care unit (ICU) [16]. The CALLY index was calculated using the formula: (Albumin g/L × Lymphocytes mm^3^)/(CRP mg/L × 10^4^) [5]. In practical terms, this composite score reflects the balance between nutritional status (albumin), systemic inflammation (CRP), and immune competence (lymphocytes), with lower values indicating poorer inflammatory–nutritional status. All blood samples were collected within 24 h of hospital admission as part of routine clinical care. Serum albumin, CRP, lymphocyte counts, and other routine biochemical parameters were measured using automated clinical chemistry analyzers. All analyses were performed in the hospital’s central clinical laboratory in accordance with standard operating procedures.

### 2.3. Statistical Analysis

The observed data of the description analyses are normally distributed (Shapiro–Wilk W-Test) and presented as means ± Standard Deviation (SD). The optimal prognostic cut-off of the CALLY index was identified by receiver operating characteristic (ROC) curve analysis. The optimal threshold for the CALLY index was identified using Youden’s J statistic on the study cohort ROC curve to balance false positives and false negatives. Diagnostic operating characteristics (AUC, sensitivity, specificity), positive predictive value (PPV), negative predictive value (NPV), and likelihood ratios (LR^+^, LR^−^) based on the observed event prevalence have been reported. Logistic regression analyses were performed to investigate the association between CALLY index and mortality after controlling for multiple covariates. Variables included in the multivariable logistic regression model were selected based on clinical relevance and prior literature, including age, sex, nutritional status, comorbidity burden, hemoglobin level, and qSOFA score. The multicollinearity among predictors has been assessed by examining variance inflation factors (VIF), and all retained variables had VIF < 2, indicating no significant multicollinearity. A survival curve was performed by Kaplan–Meier. All *p* values are two-tailed, and the level of significance was set at *p*  ≤  0.05. Statistical analyses were performed using the SPSS 26 software package (SPSS, Inc., Chicago, IL, USA).

## 3. Results

### 3.1. A Descriptive Analysis of the Study Population

A total of 264 patients were enrolled in the study, comprising 167 women (63.3%) and 97 men (36.7%), all of whom were admitted to the acute geriatric care unit. The mean age of the cohort was 88.02 ± 6.37 years. Men were significantly younger than women (86.98 ± 6.07 vs. 88.62 ± 6.47, *p* = 0.044) and had lower total cholesterol (130.14 ± 36.31 vs. 147.42 ± 45.11; *p* = 0.023) as well as higher blood creatinine value (1.41 ± 0.83 vs. 1.10 ± 0.66; *p* = 0.001). The main characteristics of the study population are summarized in Table 1.

The most frequent reasons for hospitalization were pneumonia and respiratory failure (*n* = 71, 26.9%), followed by sepsis (*n* = 29, 11.0%), delirium (*n* = 28, 10.6%), falls and postural instability (*n* = 27, 10.2%), heart failure (*n* = 24, 9.1%), bowel obstruction (*n* = 13, 4.9%), and stroke (*n* = 12, 4.5%). The remaining 22.4% of patients were admitted for various other acute medical conditions.

### 3.2. CALLY Index, Clinical and Biochemical Parameters

A significant correlation was found between the CALLY index and some clinical parameters, including ADL (r = 0.178; *p* = 0.005), IADL (r = 0.167; *p* = 0.008), systolic blood pressure (r = 0.173; *p* = 0.005), diastolic blood pressure (r = 0.124; *p* = 0.004), quick sequential organ failure assessment (r = −0.154; *p* = 0.013), and total cholesterol (r = 0.192; *p* = 0.027). After adjusting for sex and age, a statistically significant association emerged exclusively between the CALLY index and hemoglobin levels (r = 0.336, *p* = 0.013).

### 3.3. CALLY Index for Short-Term Mortality

**Figure 2:**
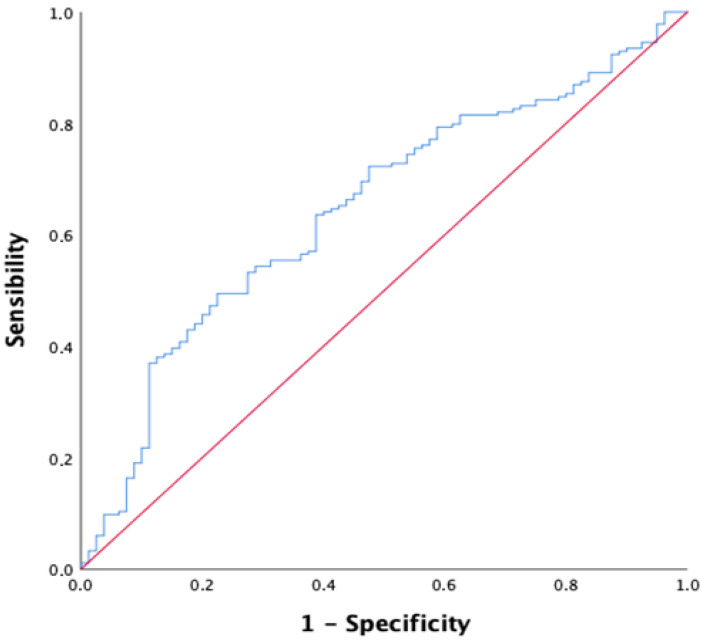
ROC curve for the CALLY index in predicting three-month mortality among patients admitted to the acute geriatric care unit. Best cut off of CALLY index; AUC = 0.647 (95% CI: 0.576–0.718; *p* < 0.001), cut-off value is: 0.055, sensitivity = 68.5% and specificity = 46.3%.

Three months following discharge from the acute care unit, a structured telephone follow-up was conducted for each participant to assess survival status. Of the 264 patients initially enrolled, 80 had died by the end of the follow-up period, corresponding to a three-month mortality rate of 30.3%. To evaluate the prognostic performance of the CALLY index in predicting mortality, a receiver operating characteristic (ROC) curve analysis was performed. The area under the ROC curve (AUC) was 0.647 (95% CI: 0.576–0.718; *p* < 0.001), indicating that while the CALLY index has statistically significant predictive value, its discriminative ability is modest (Figure 2).

The optimal cut-off value for survival was chosen at 0.055, thus providing an acceptable trade-off (sensitivity 68.5%, specificity 46.3%). Based on the optimal cut-off value (0.055) identified through ROC curve analysis, the study population was stratified into two groups: patients with a CALLY index < 0.055 (*n* = 101, 38.3%) and those with a CALLY index ≥ 0.055 (*n* = 163, 61.7%). Individuals in the lower CALLY index group (<0.055) demonstrated significantly poorer functional status, as evidenced by reduced ADL scores. Biochemically and hemodynamically, they also presented with significantly lower total cholesterol, systolic, and diastolic blood pressure values. Conversely, this group exhibited significantly higher qSOFA scores. The principal clinical, functional, and biochemical characteristics of the two groups are detailed in Table 2.

Contingency analyses revealed a marked difference in three-month mortality rates between the two CALLY index groups. Specifically, 42.5% of patients with a CALLY index < 0.055 (43 out of 101) died within the follow-up period, compared to only 22.6% (37 out of 163) in the group with a CALLY index ≥ 0.055. The operating characteristics at this threshold were: PPV 53.7% (43/80), NPV 77.3% (126/163), LR^+^ 1.28, and LR^−^ 0.68.

To further elucidate the relationship between CALLY index and short-term mortality, a multivariate logistic regression analysis was conducted (Table 3).

**Figure 3:**
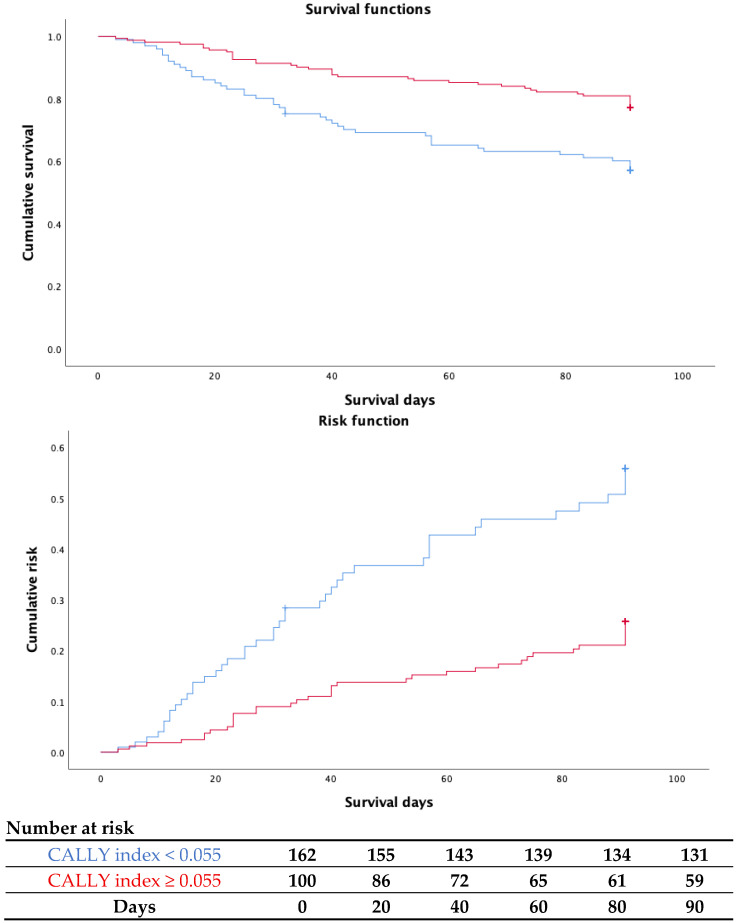
Cumulative survival and risk functions stratified by CALLY index scores. Red: CALLY index ≥ 0.055; Blue: CALLY index < 0.055.

After controlling for potential confounders—including age, sex, comorbidity burden (CIRS-G), nutritional status (MNA), hemoglobin levels, and qSOFA score—a lower CALLY index remained independently associated with increased mortality (B = −0.805; OR = 0.45; 95% CI: 0.215–0.930; *p* = 0.031). Finally, Kaplan–Meier survival analysis was performed to evaluate differences in cumulative survival based on CALLY index stratification. As illustrated in Figure 3, patients with a CALLY index ≥ 0.055 demonstrated significantly longer survival compared to those with index values < 0.055. The survival difference between the two groups was statistically confirmed by the Mantel–Cox log-rank test (χ^2^ = 13.71; *p* < 0.001), further underscoring the prognostic relevance of the CALLY index in this geriatric cohort.

## 4. Discussion

In this study, conducted in a cohort of very old, hospitalized patients characterized by multimorbidity and functional decline, we found that (1) the CALLY index is closely associated with key clinical, functional, and biochemical indicators of vulnerability, and (2) a low CALLY index independently predicts higher short-term mortality, thereby identifying patients at greatest risk after acute hospitalization.

The CALLY index, which integrates serum albumin, CRP, and lymphocyte count, provides a multidimensional assessment of nutritional, inflammatory, and immune status. Serum albumin is a key biomarker of nutritional status, with reduced levels commonly observed in patients with malnutrition [17]. However, inflammatory processes can decrease serum albumin levels independently of nutritional status [18]. Therefore, relying solely on serum albumin to assess nutritional status is insufficient and should be complemented by evaluation of the patient’s immune and inflammatory profile. Lymphocytes are an important component in assessing the body’s immune function [19]. However, they are also widely recognized as indirect indicators of nutritional status [20]. In older adults, low lymphocyte counts have consistently been associated with sarcopenia [21], frailty [22], and increased mortality [23], reflecting a compromised nutritional [20] and immunological reserve [19]. Therefore, including lymphocyte count in the CALLY index strengthens its biological relevance by linking systemic inflammation and nutritional status with immune function, three critical determinants of outcomes in geriatric patients. Elevated CRP levels are strongly associated with catabolic processes, muscle wasting, and functional decline in older adults, and have been shown to predict adverse outcomes, including frailty, disability, and mortality [24]. Incorporating CRP into the CALLY index thus enhances its prognostic power by capturing the inflammatory component that often drives the progression of malnutrition and immune dysfunction in geriatric populations.

Our study shows that individuals in the lower CALLY index group have significantly poorer functional status, as evidenced by reduced ADL scores. They also presented with significantly lower systolic and diastolic blood pressure values as well as lower total cholesterol while higher qSOFA scores. These data suggest that the CALLY index reflects not only biochemical markers of inflammation and nutrition but also the functional and physiological reserve that are critical determinants of prognosis in geriatric patients. The independent correlation between the CALLY index and hemoglobin after adjusting for age and sex suggests that both may reflect shared physiological pathways involving chronic disease burden, nutritional deficiency, and systemic inflammation.

Although it was initially validated as a diagnostic and prognostic tool in various malignancies, CALLY index utility may extend to geriatric populations, in whom chronic low-grade inflammation, malnutrition, and immunosenescence represent hallmark features of aging. Accordingly, a recent study performed in both community-dwelling and hospitalized older adults revealed that higher CALLY index values correlate with a lower likelihood of sarcopenia, suggesting its potential as a simple, clinically useful marker for early risk assessment in aging populations [25]. Another study was recently conducted in a community-based, observational setting, utilizing data from the National Health and Nutrition Examination Survey (NHANES), a large, nationally representative cohort of older adults (≥ 60 years) in the United States. This population-level design allowed for the evaluation of the prognostic value of the CALLY index in predicting cardiovascular mortality among community-dwelling older individuals [26]. Furthermore, a national cohort study established the applicability of the CALLY index for monitoring adverse outcomes in community-dwelling older adult populations in China [11].

The CALLY index shows modest discrimination (AUC = 0.647) with LR^+^: 1.28 and LR^−^: 0.68, values that are insufficient for stand-alone decision-making. Clinically, this profile suggests that CALLY is more useful as a triage adjunct for ruling-out very high short-term risk (NPV: 77% at the chosen cut-off) than for confidently ruling-in mortality risk (PPV: 53.7%). Its strengths are simplicity, availability, and biological interpretability (integration of inflammation, nutrition, and immune competence). Multivariate logistic regression analysis demonstrates that a CALLY index < 0.055 was independently associated with an increased risk of 3-month mortality after hospital discharge, indicating that patients with higher CALLY values had approximately a 55% lower risk of death. Again, higher nutritional status, as reflected by the MNA score, is also associated with a lower mortality risk.

Our data align with previous research demonstrating the CALLY index’s prognostic value in various clinical settings. For instance, a recent study reported that higher CALLY index values correlated with significantly lower 30-day and 60-day mortality rates in patients with sepsis [6]. Similarly, a study on older patients with heart failure with preserved ejection fraction (HFpEF) highlighted the index as a novel long-term prognostic marker, emphasizing its relevance in populations where chronic inflammation and malnutrition exacerbate adverse outcomes [7]. From a clinical perspective, incorporating the CALLY index into routine geriatric assessments could support early identification of high-risk patients, inform nutritional and functional interventions, and guide discharge planning or post-discharge surveillance strategies.

Some limitations of this study must be acknowledged. First, its retrospective, single-center design may limit generalizability. Second, the absence of external validation means that the prognostic performance of the CALLY index should be confirmed in independent cohorts. Third, the modest discriminative accuracy observed underscores that while the index is informative, it should be integrated with other clinical and functional assessments. Fourth, we did not evaluate dynamic changes in the CALLY index over time, which could provide additional prognostic insight. Finally, no direct comparison was made with established prognostic tools such as the Geriatric Nutritional Risk Index (GNRI) [27] or Controlling Nutritional Status (CONUT) score [28], which would be valuable for contextualizing its clinical utility. A further limitation is the lack of detailed information on the causes of death. As many patients died at home and follow-up was conducted by telephone, it was not possible to reliably ascertain whether mortality was directly related to the acute illness, to chronic comorbidities, or to other unrelated events. Future research should focus on multicenter, prospective validation of the CALLY index in diverse geriatric populations. Comparative studies against established prognostic models and analyses incorporating serial biomarker measurements may further clarify its role in clinical decision-making and enhance its predictive power.

## 5. Conclusions

In conclusion, the CALLY index captures a multidimensional vulnerability profile by integrating nutritional, inflammatory, and immune components, offering incremental prognostic information beyond conventional measures. While its discriminative accuracy is modest, its simplicity, availability, and low cost make it a promising adjunct to established risk stratification tools, supporting early nutritional intervention, intensified monitoring, and individualized care planning in acute geriatric medicine. Future multicenter prospective studies are needed to confirm these findings.

## Figures and Tables

**Table 1:** Demographic, clinical, multidimensional, and biochemical characteristics of the study population (*n* = 264), stratified by sex.

	All (n = 264)	Women (n = 167)	Men (n = 97)	*p*
Age (years)	88.02 ± 6.37	88.62 ± 6.47	86.98 ± 6.07	0.044
Activities of Daily Living (ADL, n)	2.19 ± 2.02	2.16 ± 1.94	2.24 ± 2.16	0.778
Instrumental ADL (IADL, n)	1.42 ± 2.03	1.37 ± 2.01	1.51 ± 2.07	0.607
Mini Nutritional Assessment (MNA, n)	17.33 ± 8.06	17.18 ± 8.62	17.58 ± 7.06	0.715
Cumulative Illness Rating Scale for Geriatrics (CIRS-G, n)	9.70 ± 5.41	9.94 ± 5.35	9.31 ± 5.51	0.419
Number of drugs (n)	7.07 ± 3.03	7.08 ± 2.99	7.05 ± 3.12	0.922
Systolic Blood Pressure (SPB, mmHg)	127.30 ± 26.93	129.36 ± 28.31	123.79 ± 24.13	0.106
Diastolic Blood Pressure (DPB, mmHg)	70.02 ± 13.34	71.12 ± 13.95	68.16 ± 12.08	0.083
Peripheral Oxygen Saturation (SpO_2_, %)	94.25 ± 11.19	94.43 ± 10.39	94.00 ± 12.33	0.814
quick Sequential Organ Failure Assessment (qSOFA, n)	1.50 ± 0.62	1.56 ± 0.62	1.42 ± 0.60	0.084
Hemoglobin (Hb, g/dL)	11.38 ± 2.05	11.31 ± 2.05	11.51 ± 2.04	0.443
White Blood Cell Count (WBC, ×10^3^/μL)	10.00 ± 6.52	9.73 ± 4.73	10.47 ± 8.79	0.373
Lymphocyte count (×10^9^/L)	1.36 ± 1.08	1.29 ± 0.69	1.48 ± 1.54	0.182
Serum Albumin (g/dL)	3.19 ± 0.47	3.21 ± 0.48	3.15 ± 0.45	0.374
C-reactive Protein (CRP, mg/dL)	6.79 ± 7.59	6.42 ± 7.03	7.42 ± 8.46	0.305
Creatinine (mg/dL)	1.21 ± 0.74	1.10 ± 0.66	1.41 ± 0.83	0.001
Glucose (mg/dL)	111.29 ± 49.73	107.06 ± 37.57	118.34 ± 64.80	0.089
Total Cholesterol (mg/dL)	140.92 ± 42.71	147.42 ± 45.11	130.14 ± 36.31	0.023
Triglycerides (mg/dL)	110.25 ± 68.22	111.66 ± 59.30	108.00 ± 81.08	0.767
CALLY index	0.60 ± 1.48	0.60 ± 1.55	0.59 ± 1.35	0.990

CALLY index: C-reactive protein–albumin–lymphocyte index.

**Table 2:** Demographic, multidimensional and biochemical characteristics of all sample population (*n* = 264), stratified by CALLY index.

	Sample Population (*n* = 264)	CALLY Index < 0.055 (*n* = 101)	CALLY Index > 0.055 (*n* = 163)	*p*
Age (years)	88.02 ± 6.37	87.85 ± 7.00	88.12 ± 5.96	0.732
Sex (M/F)	97/167	40/61	57/106	0.448
Activities of Daily Living (ADL, *n*)	2.19 ± 2.02	1.74 ± 1.88	2.46 ± 2.07	0.007
Instrumental ADL (IADL, *n*)	1.42 ± 2.03	1.11 ± 1.75	1.60 ± 2.16	0.064
Mini Nutritional Assessment (MNA, *n*)	18.10 ± 14.24	16.12 ± 8.43	18.05 ± 7.77	0.079
Cumulative Illness Rating Scale for Geriatrics (CIRS-G, *n*)	9.70 ± 5.41	9.78 ± 5.70	9.66 ± 5.26	0.874
Number of drugs (*n*)	7.07 ± 3.03	6.80 ± 3.19	7.24 ± 2.93	0.250
Systolic Blood Pressure (SPB, mmHg)	127.30 ± 26.93	116.41 ± 21.24	133.92 ± 27.90	<0.001
Diastolic Blood Pressure (DPB, mmHg)	70.02 ± 13.34	66.33 ± 11.47	72.26 ± 13.93	<0.001
Peripheral Oxygen Saturation (SpO_2_, %)	94.25 ± 11.19	93.08 ± 13.78	94.82 ± 9.73	0.372
Quick Sequential Organ Failure Assessment (qSOFA, *n*)	1.50 ± 0.62	1.71 ± 0.71	1.38 ± 0.52	<0.001
Hemoglobin (Hb, g/dL)	11.38 ± 2.05	11.17 ± 2.02	11.52 ± 2.05	0.173
White Blood Cell Count (WBC, ×10^3/^μL)	10.00 ± 6.52	11.12 ± 5.55	9.31 ± 6.98	0.028
Lymphocyte count (×10^9^/L)	1.36 ± 1.08	0.98 ± 0.51	1.59 ± 1.26	<0.001
Serum Albumine (g/dL)	3.19 ± 0.47	2.94 ± 0.46	3.34 ± 0.41	<0.001
C-reactive Protein (CRP, mg/dL)	6.79 ± 7.59	13.98 ± 7.46	2.34 ± 2.65	<0.001
Creatinine (mg/dL)	1.21 ± 0.74	1.30 ± 0.84	1.16 ± 0.67	0.137
Glucose (mg/dL)	111.29 ± 49.73	116.50 ± 50.14	107.99 ± 49.36	0.197
Total Cholesterol (mg/dL)	140.92 ± 42.71	127.50 ± 34.68	148.02 ± 44.98	0.008
Triglycerides (mg/dL)	110.25 ± 68.22	117.20 ± 70.77	106.57 ± 66.96	0.400

CALLY index: C-reactive protein–albumin–lymphocyte index.

**Table 3:** Multivariate logistic regression analysis for 3-month mortality (*n* = 264).

Variable	B	OR	95% CI	*p*-Value
Age (years)	0.053	1.055	0.99–1.12	0.087
Sex (F vs. M)	−0.310	0.733	0.36–1.51	0.400
Number of drugs	0.067	1.069	0.94–1.21	0.294
CIRS-G	0.041	1.042	0.98–1.11	0.185
MNA	−0.050	0.952	0.91–0.99	0.030
Hemoglobin (g/dL)	0.052	1.053	0.88–1.26	0.577
qSOFA	0.312	1.367	0.75–2.49	0.309
CALLY index (<0.055)	−0.805	0.447	0.22–0.93	0.031

B indicates the logistic regression coefficient (log-odds); OR: odds ratio; CI: confidence interval; CIRS-G: Cumulative Illness Rating Scale for Geriatrics; MNA: Mini Nutritional Assessment; qSOFA: quick Sequential Organ Failure Assessment. Model summary: Nagelkerke R^2^ = 0.172.

## Data Availability

The data presented in this study are available on request from the corresponding author due to privacy and ethical reasons.
